# Supplementary material for: Chlorpromazine eliminates acute myeloid leukemia cells by perturbing subcellular localization of FLT3-ITD and KIT-D816V
Source: Nat Commun. 2020 Aug 18;11:4147. doi: 10.1038/s41467-020-17666-8 (PMC7434901; doi:10.1038/s41467-020-17666-8)
Supplement: Supplementary file 1 — Supplementary Information [file 41467_2020_17666_MOESM1_ESM.pdf]

Supplementary Information for

Chlorpromazine eliminates acute myeloid leukemia cells by perturbing subcellular localization of FLT3-ITD and KIT-D816V

Shinya Rai, et al.

*Corresponding author. Email: htanaka@med.kindai.ac.jp

The PDF file includes:

Supplementary Figure 1: *CALM* shRNA perturbs signals from MT-RTKs in leukemia cells.

Supplementary Figure 2: CPZ treatment induces apoptosis in Ba/F3 cells with MT-RTKs.

Supplementary Figure 3: CD34^{+}38^{-} fraction contains human AML initiating cells.

Supplementary Figure 4: CALM depletion perturbs subcellular localization of MT-RTKs.

Supplementary Figure 5: Plasm concentration of CPZ in mice treated with 10mg/kg CPZ.

Supplementary Figure 6: CPZ is still effective for already developed AML mice model.

Supplementary Figure 7: CPZ doesn't affect capacity of normal hematopoietic cells.

Supplementary Figure 8: Gating strategies used for cell sorting and analysis with FACS

Supplementary Figure 9: Uncropped versions of immunoblots analyses.

Supplementary Table 1: Clinical characteristics of patients.

Supplementary Table 2: List of antibodies used in this study.

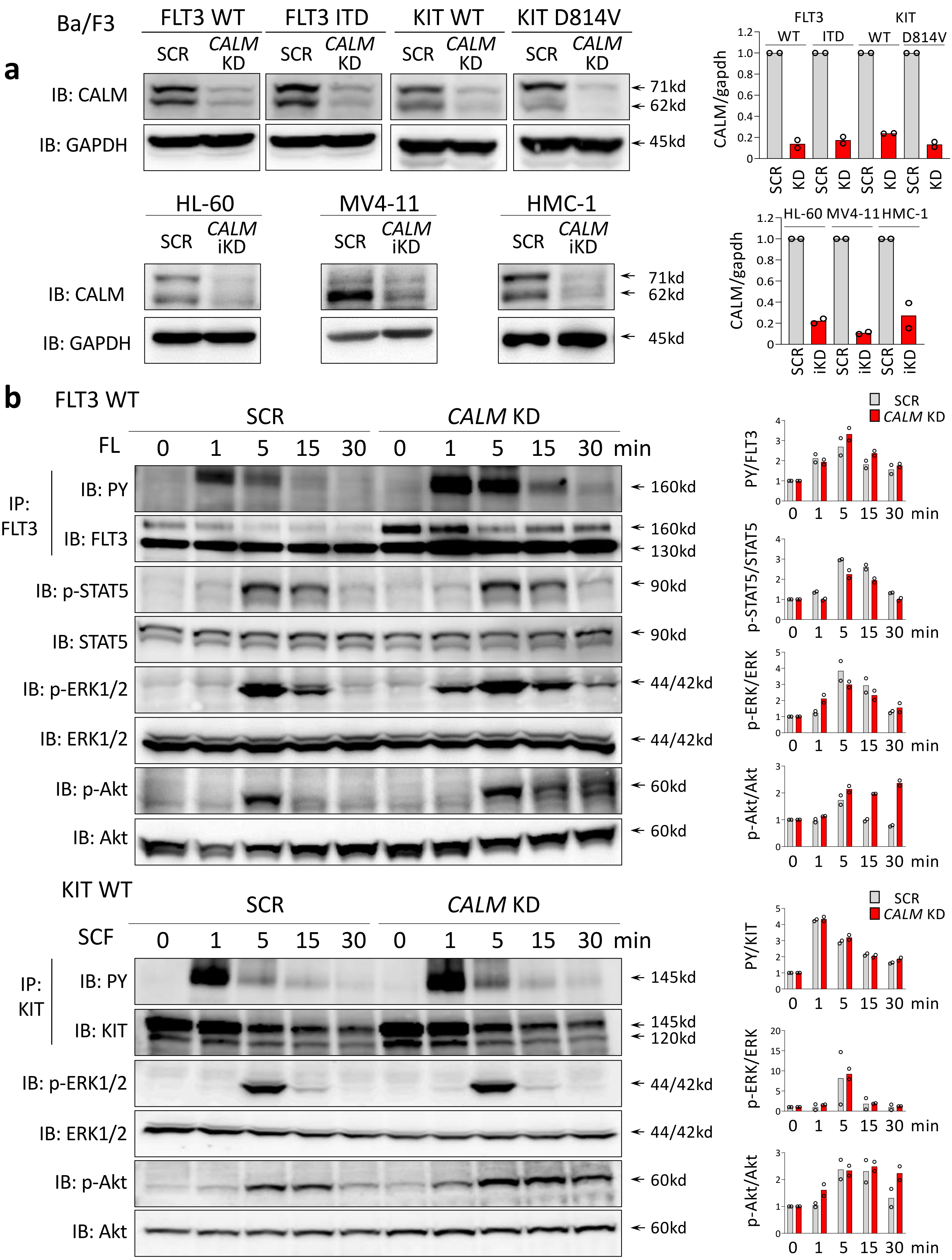
a
Ba/F3
FLT3 WT
FLT3 ITD
KIT WT
KIT D814V
SCR
CALM KD
IB: CALM
IB: GAPDH
71kd
62kd
45kd
FLT3
KIT
WT
ITD
D814V
CALM/gapdh
SCR
KD
HL-60
MV4-11
HMC-1
CALM iKD
iKD
b
FLT3 WT
FL
0 1 5 15 30 min
IP: FLT3
IB: PY
IB: FLT3
IB: p-STAT5
IB: STAT5
IB: p-ERK1/2
IB: ERK1/2
IB: p-Akt
IB: Akt
160kd
130kd
90kd
44/42kd
60kd
PY/FLT3
p-STAT5/STAT5
p-ERK/ERK
p-Akt/Akt
KIT WT
SCF
IP: KIT
IB: KIT
145kd
120kd
PY/KIT

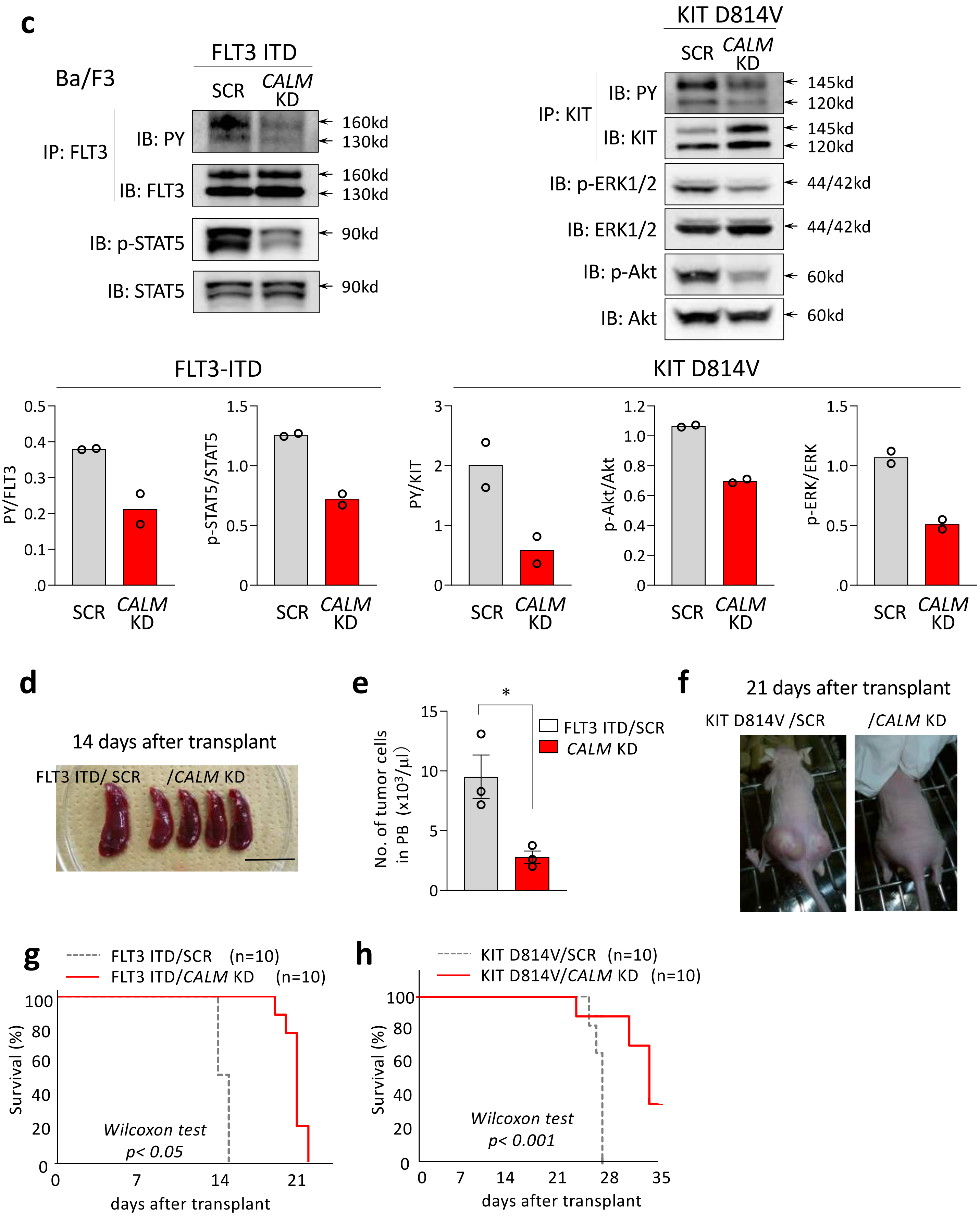

c
Ba/F3
FLT3 ITD
SCR
CALM KD
IP: FLT3
IB: PY
IB: FLT3
IB: p-STAT5
IB: STAT5
160kd
130kd
160kd
130kd
90kd
90kd
KIT D814V
SCR
CALM KD
IP: KIT
IB: PY
IB: KIT
IB: p-ERK1/2
IB: ERK1/2
IB: p-Akt
IB: Akt
145kd
120kd
145kd
120kd
44/42kd
44/42kd
60kd
60kd
FLT3-ITD
KIT D814V
PY/FLT3
p-STAT5/STAT5
PY/KIT
p-Akt/Akt
p-ERK/ERK
SCR
CALM KD
d
14 days after transplant
FLT3 ITD/ SCR
/CALM KD
e
*
No. of tumor cells in PB (x10³/µl)
FLT3 ITD/SCR
CALM KD
f
21 days after transplant
KIT D814V /SCR
/CALM KD
g
FLT3 ITD/SCR (n=10)
FLT3 ITD/CALM KD (n=10)
Survival (%)
Wilcoxon test
p< 0.05
days after transplant
h
KIT D814V/SCR (n=10)
KIT D814V/CALM KD (n=10)
Survival (%)
Wilcoxon test
p< 0.001
days after transplant

Supplementary Figure 1

***CALM* shRNA perturbs signals from MT-RTKs in leukemia cells.**

(a) Immunoblot blot analysis was conducted to assess the expression levels of CALM protein in the indicated cells. Densitometry analyses was carried out by Image Quant TL, and data is from two independent experiments.

(b) Ba/F3-FL3 WT/*CALM* KD and /SCR cells were stimulated with 100 ng/ml of FL for up to 30 min. Changes in tyrosine phosphorylation of FLT3, STAT5, ERK1/2, and Akt were compared between these clones by immunoblot analyses using the indicated Abs. Also, changes in tyrosine phosphorylation of KIT, ERK1/2, and Akt were examined after stimulation with 100 ng/ml of SCF in Ba/F3-KIT WT/KD and /SCR cells. Densitometry analyses was carried out by Image Quant TL, and data is from two independent experiments.

(c) Tyrosine phosphorylation of FLT3 ITD, KIT D814V, and their downstream molecules (STAT5, ERK1/2, and Akt) was investigated by immunoblot analyses in the indicated clones. Densitometry analyses was carried out by Image Quant TL, and data is from two independent experiments.

(d) Ba/F3-FLT3 ITD/*CALM* KD or Ba/F3-FLT3 ITD/SCR cells (each 5×10^3 cells/100μl PBS) were injected into the tail veins of BALB/C mice. These mice were euthanized 14 days after transplantation. Specimens were collected and subjected to analyses. Representative photographs of spleens isolated from the indicated recipient mice are shown. The scale bar represents 1 cm.

(e) GFP-labelled Ba/F3-FLT3 ITD/*CALM* KD or Ba/F3-FLT3 ITD/SCR cells were transplanted into BALB/C mice. The percentage of GFP-positive cells in the peripheral blood (PB) were analyzed by flow cytometry 14 days after transplantation. The results are shown mean ± SEM from three independent experiments. Two-sided unpaired Student's t-test, *p=0.0235.

(f) Ba/F3-KIT D814V/*CALM* KD and Ba/F3-KIT D814V/SCR (2×10^6 cells/100μl PBS) were subcutaneously injected at two sites in 2.5 Gy-irradiated nude mice. These mice were carefully monitored for visible tumors at the sites of injection. Representative photographs of tumor bearing mice are shown.

(g)(h) Survival of mice transplanted with 1×10^4 Ba/F3-FLT3 ITD/*CALM* KD and /SCR cells were compared by Kaplan Maier analysis (upper panel) (each group n=10, P =2.03511E-06). Similar analyses were conducted with Ba/F3-KIT D814V/*CALM* KD and /SCR cells (each group n=10, P =0.0343). Two-sided Wilcoxon test.

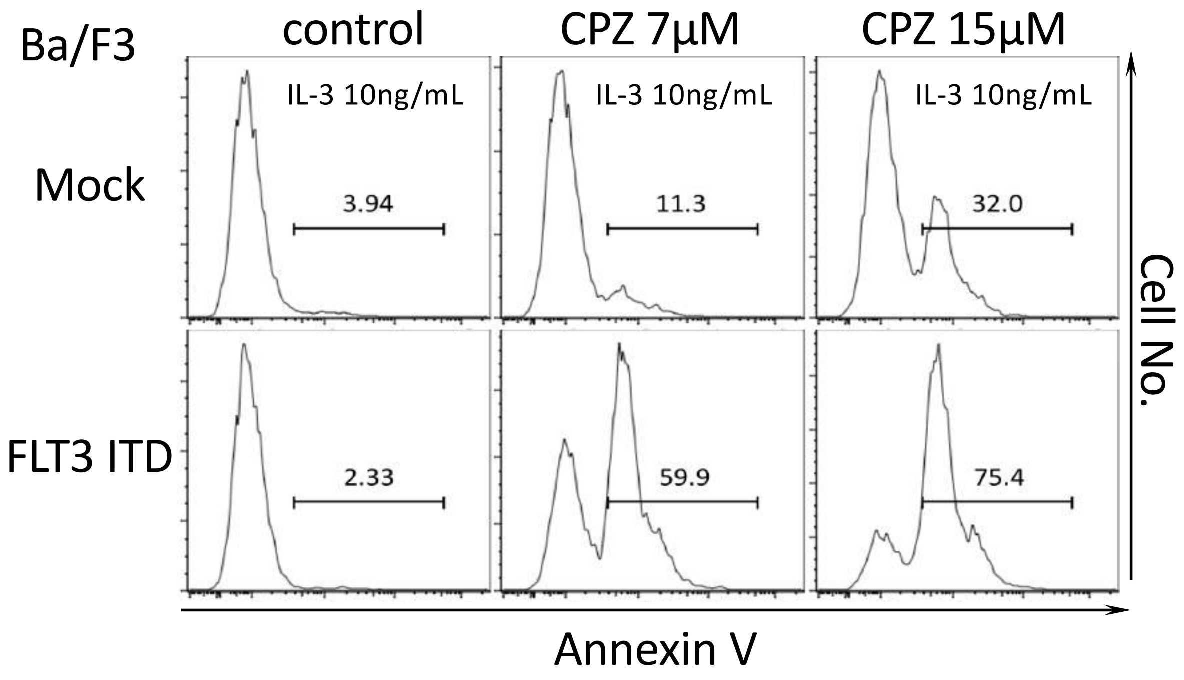
Ba/F3
control
CPZ 7μM
CPZ 15μM
IL-3 10ng/mL
IL-3 10ng/mL
IL-3 10ng/mL
Mock
3.94
11.3
32.0
FLT3 ITD
2.33
59.9
75.4
Cell No.
Annexin V

Supplementary Figure 2

CPZ treatment induces apoptosis in Ba/F3 cells with MT-RTKs.

The effects of CPZ on IL-3-dependent and FLT ITD-dependent survival of Ba/F3 cells were evaluated by flow cytometry. Apoptotic cells were detected as Annexin V-positive cells. The proportions of apoptotic cells are indicated in each figure.

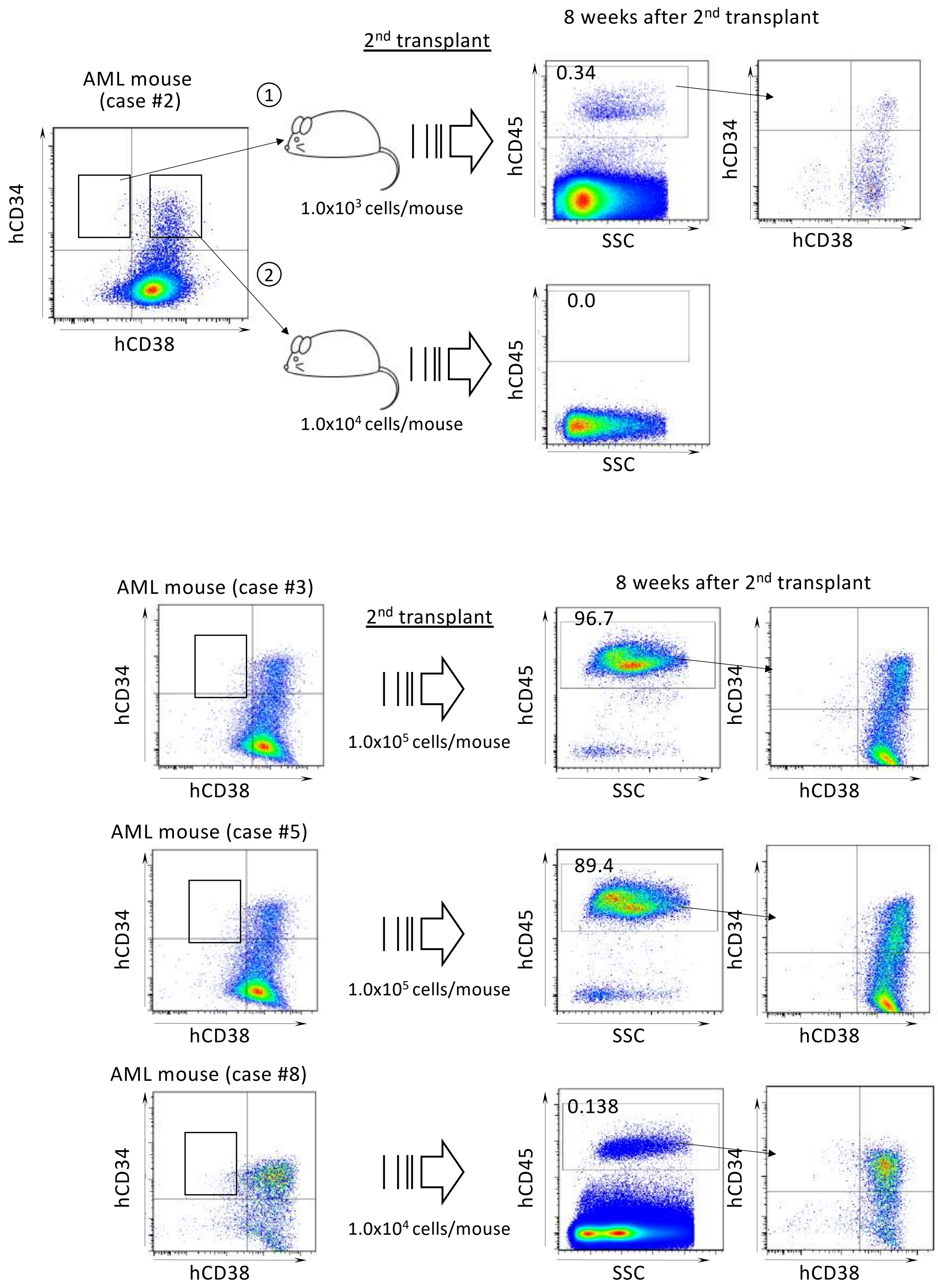

8 weeks after 2nd transplant
2nd transplant
AML mouse
(case #2)
①
②
hCD34
hCD38
1.0x10³ cells/mouse
1.0x10⁴ cells/mouse
0.34
0.0
hCD45
SSC
AML mouse (case #3)
1.0x10⁵ cells/mouse
96.7
AML mouse (case #5)
1.0x10⁵ cells/mouse
89.4
AML mouse (case #8)
1.0x10⁴ cells/mouse
0.138

Supplementary Figure 3

CD34+38- fraction contains human AML initiating cells.

After the 1st transplantation of BM cells from AML cases #2, #3, #5, and #8, all transplanted NOG mice developed AML. ①$1 \times 10^3$ $hCD34^+hCD38^-$ cells or ②$1 \times 10^4$ $hCD34^+CD38^+$ cells were isolated and transplanted into the 2nd recipient NOG mice. Eight weeks after the 2nd transplantation, BM cells were isolated from these mice and subjected to flow cytometric analyses using anti-hCD45, hCD34, and hCD38 Abs.

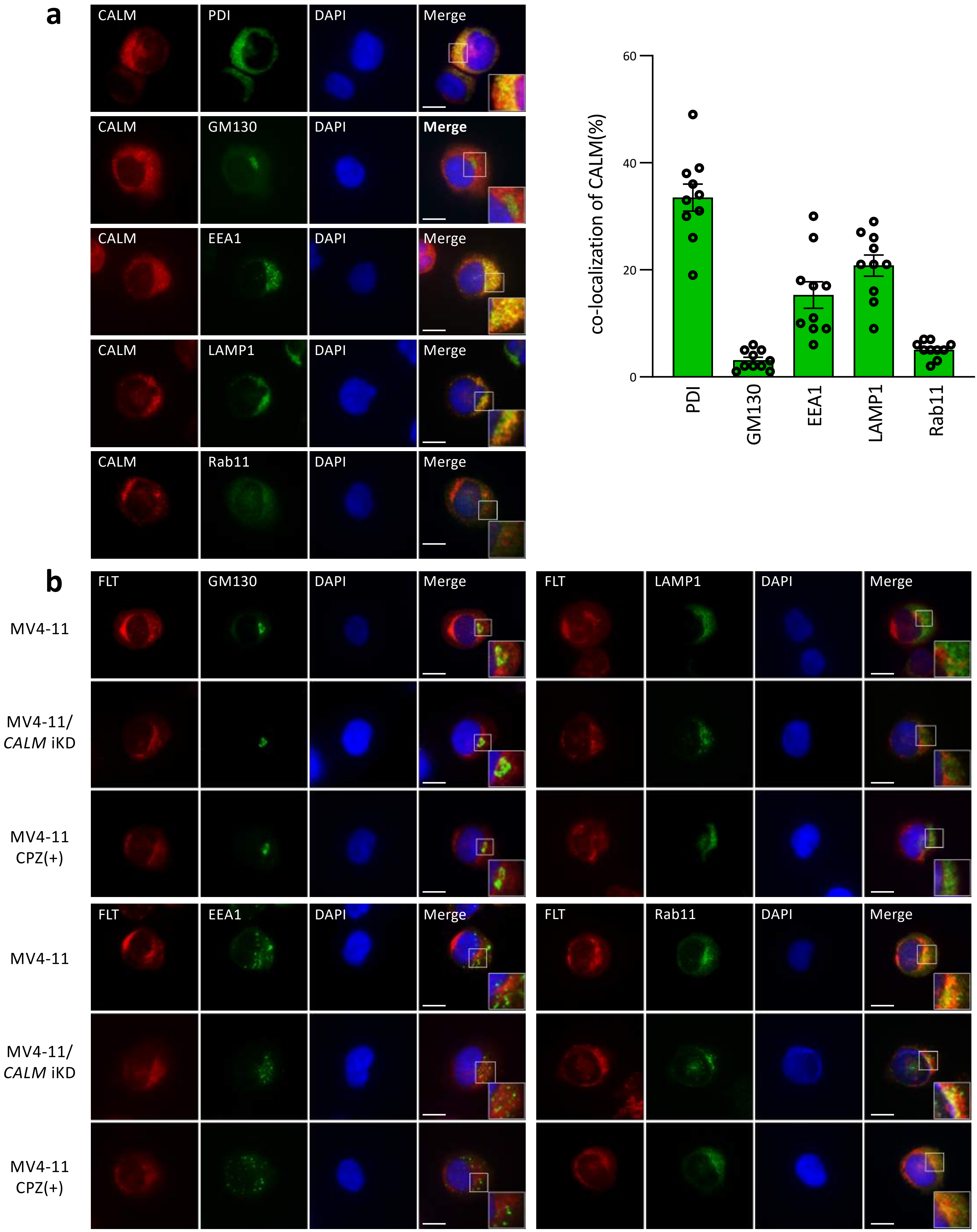
a
CALM
PDI
DAPI
Merge
GM130
EEA1
LAMP1
Rab11
co-localization of CALM(%)
60
40
20
0
PDI
GM130
EEA1
LAMP1
Rab11
b
FLT
GM130
DAPI
Merge
LAMP1
EEA1
Rab11
MV4-11
MV4-11/ CALM iKD
MV4-11 CPZ(+)

c

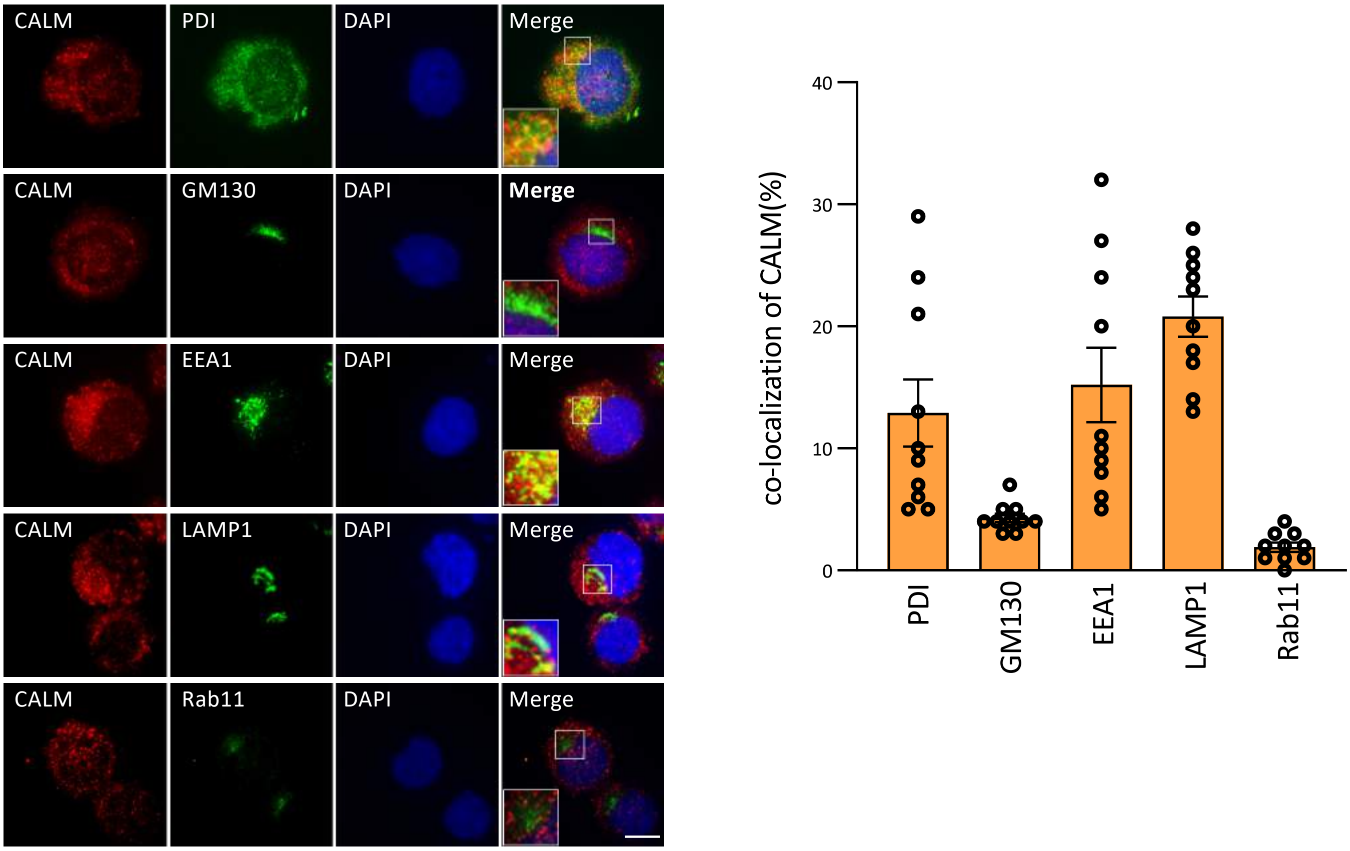

d

HMC-1

HMC-1/
CALM iKD

HMC-1
CPZ(+)

HMC-1

HMC-1/
CALM iKD

HMC-1
CPZ(+)

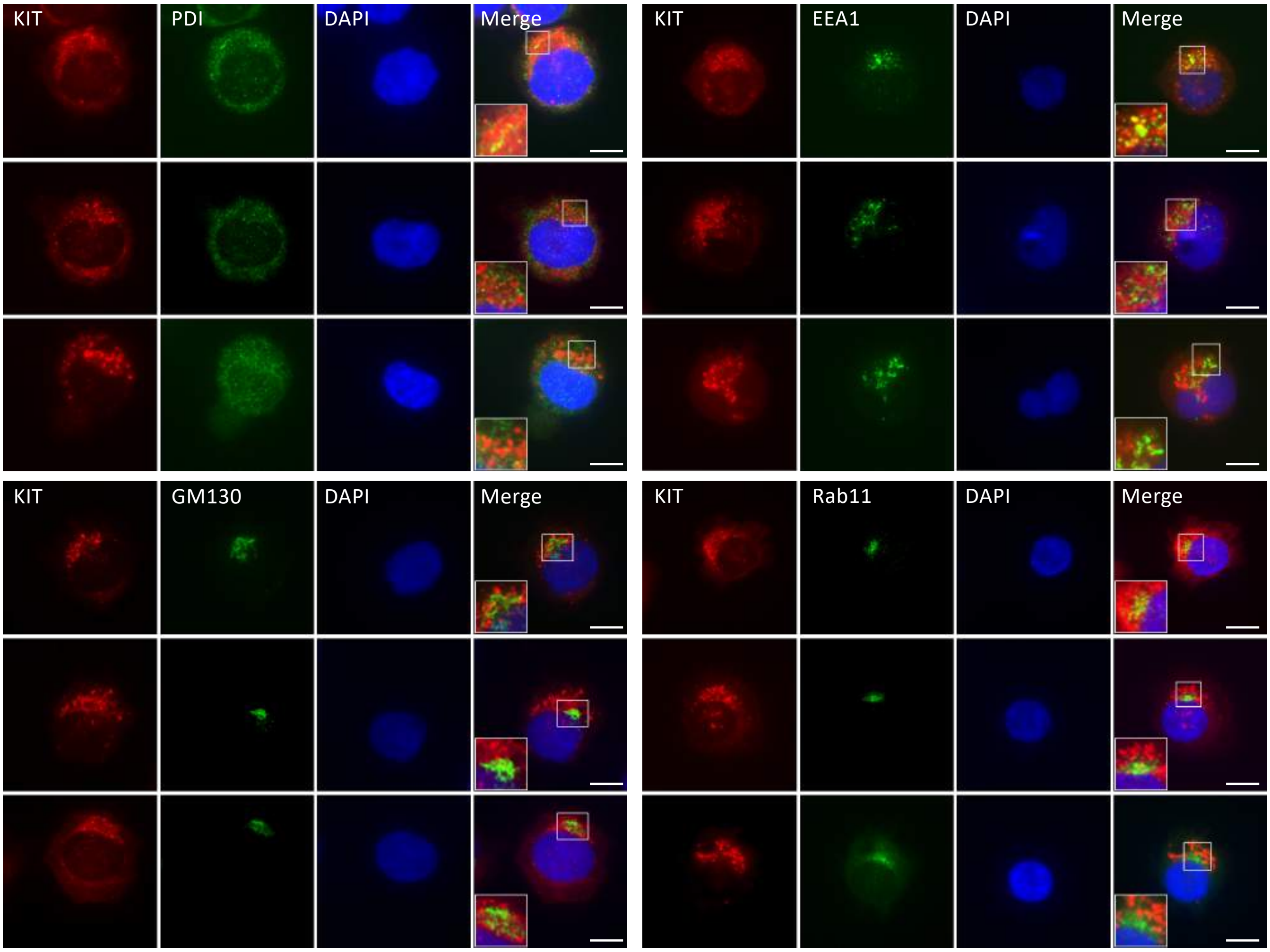

Supplementary Figure 4

CALM depletion perturbs subcellular localization of MT-RTKs.

(a) Parental MV4-11 cells were subjected to immunofluorescence microscopy. The cells were co-stained with anti-CALM Ab (red), anti-PDI Ab, anti-GM130 Ab, anti-EEA1 Ab, anti-LAMP1 Ab, anti-Rab11 Ab (green), and DAPI (blue). Scale bars, 20μm. The right lower panels show regions with higher magnification. The graph bars show the percentages of co-localization of CALM with PDI, GM130, EEA1, LAMP1, and Rab11 in Parental MV4-11 cells. Results (%) represent the means ± SEM from 10 cells.

(b) Parental, *CALM* iKD, and 18-h CPZ-treated MV4-11 cells were co-stained with the anti-FLT3 Ab (red), anti-GM130 Ab, anti-EEA1 Ab, anti-LAMP1 Ab, anti-Rab11 Ab (green), and DAPI (blue). Scale bars, 20 μm. The right lower panels show regions with higher magnification.

(c) Parental HMC-1 cells were subjected to immunofluorescence microscopy. The cells were co-stained with anti-CALM Ab (red), anti-PDI Ab, anti-GM130 Ab, anti-EEA1 Ab, anti-LAMP1 Ab, anti-Rab11 Ab (green), and DAPI (blue). Scale bars, 20μm. The left lower panels show regions with higher magnification. The graph bars show the percentages of co-localization of CALM with PDI, GM130, EEA1, LAMP1, and Rab11 in Parental HMC-1 cells. Results (%) represent the means ± SEM from 10 cells.

(d) Parental, *CALM* iKD, and 24-h CPZ-treated HMC-1 cells were co-stained with the anti-KIT Ab (red), anti-PDI Ab, anti-GM130 Ab, anti-EEA1 Ab, anti-Rab11 Ab (green), and DAPI (blue). Scale bars, 20 μm. The left lower panels show regions with higher magnification.

These images are representative of three independent experiments.

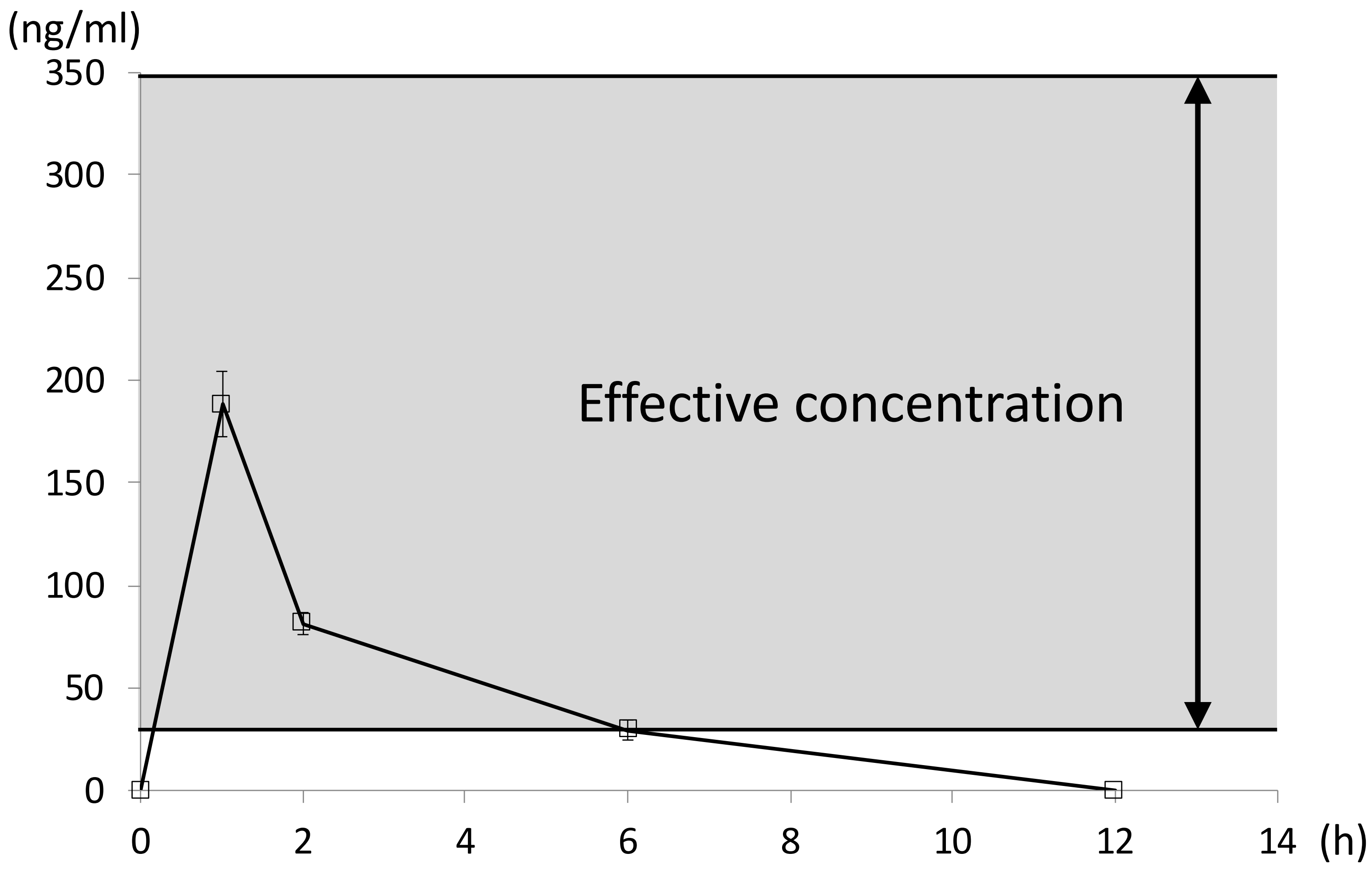
(ng/ml)
350
300
250
200
150
100
50
0
0
2
4
6
8
10
12
14
(h)
Effective concentration

Supplementary Figure 5

Plasm concentration of CPZ in mice treated with 10mg/kg CPZ.

CPZ was administered intraperitoneally into 6-8-week old female BALB/C mice at 10 mg/kg. Plasma samples were harvested at 0, 1, 2, 6, 12 h after the intraperitoneal administration of CPZ (n=12). Effective blood concentration of CPZ as an antipsychotic drug ranges from 30 to 350 ng/ml as previously reported. The X axis indicate the time (h), and the Y axis indicate plasma concentration of CPZ (ng/ml). The results of each point indicate the mean ± SEM from three independent mice.

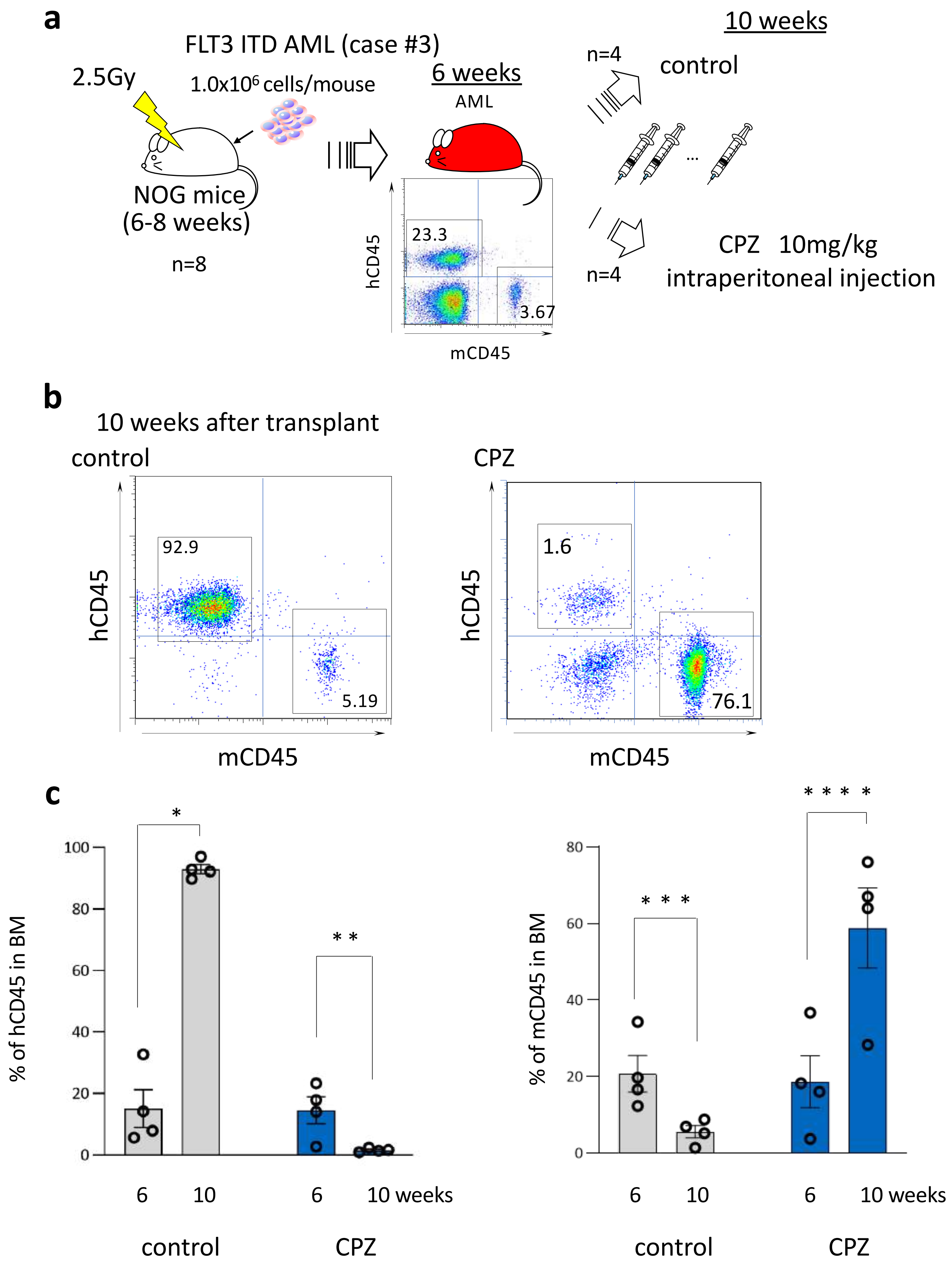
a
FLT3 ITD AML (case #3)
2.5Gy
1.0x10⁶ cells/mouse
NOG mice
(6-8 weeks)
n=8
6 weeks
AML
23.3
3.67
hCD45
mCD45
10 weeks
n=4
control
n=4
CPZ 10mg/kg
intraperitoneal injection
b
10 weeks after transplant
control
92.9
5.19
CPZ
1.6
76.1
hCD45
mCD45
c
% of hCD45 in BM
% of mCD45 in BM
6
10
10 weeks
control
CPZ

Supplementary Figure 6

CPZ is still effective for already developed AML mice model.

(a) Schematic representation of the xenotransplantation model and subsequent treatment utilized. Six weeks after transplantation with FLT3 ITD$^+$ AML cells, all transplanted NOG mice developed AML, which was confirmed by flow cytometric analyses of BM samples (n=4; a representative result is shown). These AML bearing mice were treated with 10 mg/kg of CPZ (n=4) or normal saline (as a control) (n=4) for 4 weeks. All control mice died of leukemia by 10 weeks after transplantation. Image is a representative figure from eight independent mice.

(b) Ten weeks after transplantation, BM samples were isolated and subjected to flow cytometric analyses using anti-hCD45 and anti-murine CD45 (mCD45) Abs. Representative results obtained from control (left panel) and CPZ-treated mice (right panel) are shown. Images are representative figure from four independent mice.

(c) The proportions of hCD45$^+$ cells (left panel) and mCD45$^+$ cells (right panel) in control mice (n=4) and CPZ-treated mice (n=4) at six and ten weeks after transplantation are shown as the mean ± SEM. Two-sided unpaired Student's t-test, **p* <0.0001, ***p*= 0.0251, ****p* =0.0235, *****p*= 0.0183.

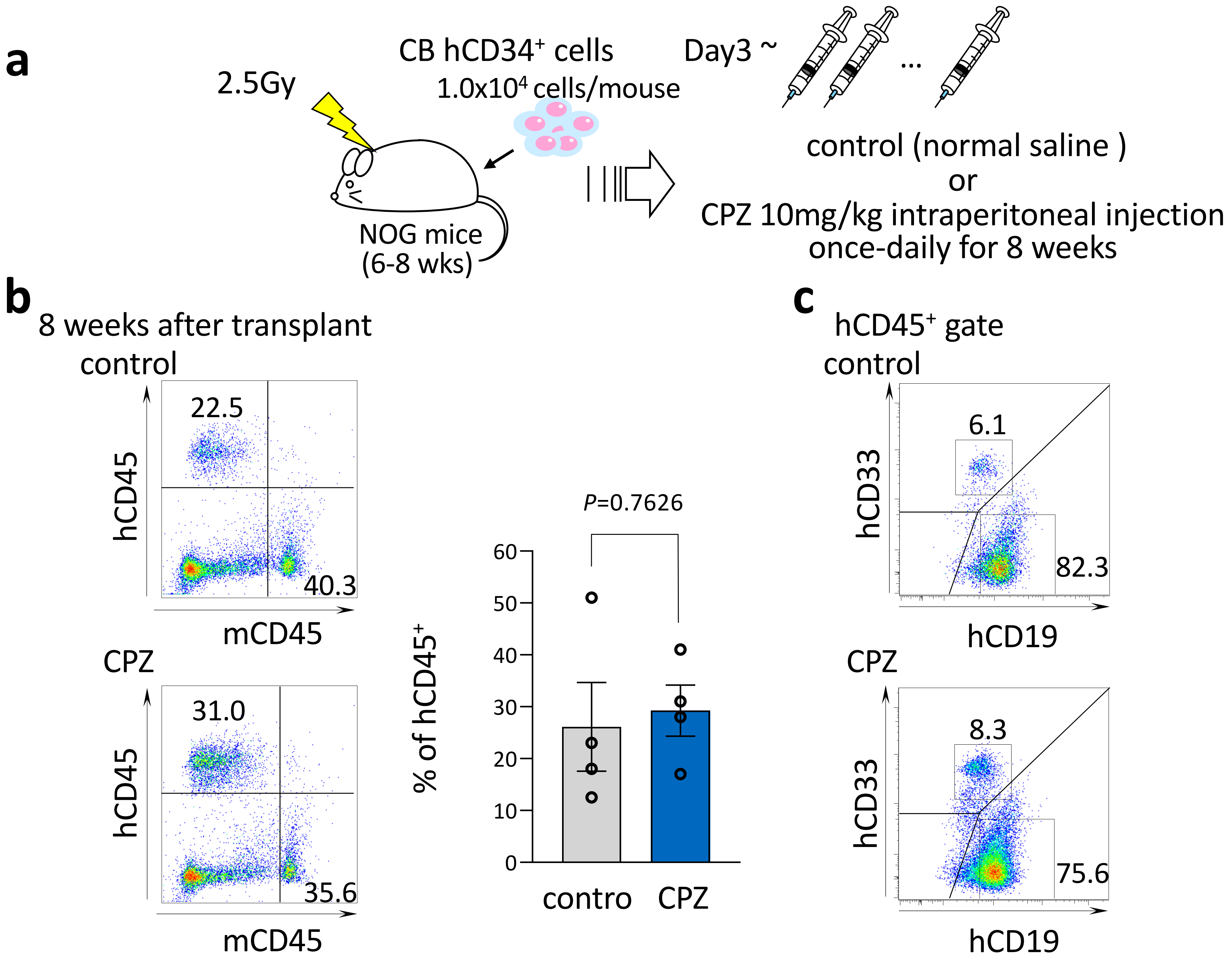
a
2.5Gy
CB hCD34+ cells
1.0x10⁴ cells/mouse
Day3 ~
...
NOG mice
(6-8 wks)
control (normal saline)
or
CPZ 10mg/kg intraperitoneal injection
once-daily for 8 weeks
b
8 weeks after transplant
control
22.5
40.3
hCD45
mCD45
CPZ
31.0
35.6
hCD45
mCD45
P=0.7626
% of hCD45+
60
50
40
30
20
10
0
contro
CPZ
c
hCD45+ gate
control
6.1
82.3
hCD33
hCD19
CPZ
8.3
75.6
hCD33
hCD19

Supplementary Figure 7

CPZ doesn't affect capacity of normal hematopoietic cells.

(a)(b) NOG mice were transplanted with 1×10^4 $CD34^+$ cells from human cord blood and treated with 10 mg/kg of CPZ or normal saline (as a control) from 3 days after transplantation. Eight weeks after transplantation, the effects of CPZ on reconstitution and repopulating capacity of transplanted normal $hCD34^+$ cells were evaluated as %$hCD45^+$ cells in BM by flow cytometry (n=4). The figures show the mean ± SEM. Two-sided unpaired Student's t-test, p=0.7626. Images are representative figure from four independent mice.

(c) The effects of CPZ on the development of $hCD45^+hCD19^+$ B cells and $hCD45^+hCD33^+$ myeloid cells from transplanted $hCD34^+$ cells were analyzed using BM samples from CPZ- and normal saline-treated mice by flow cytometry. Images are representative figure from four independent mice.

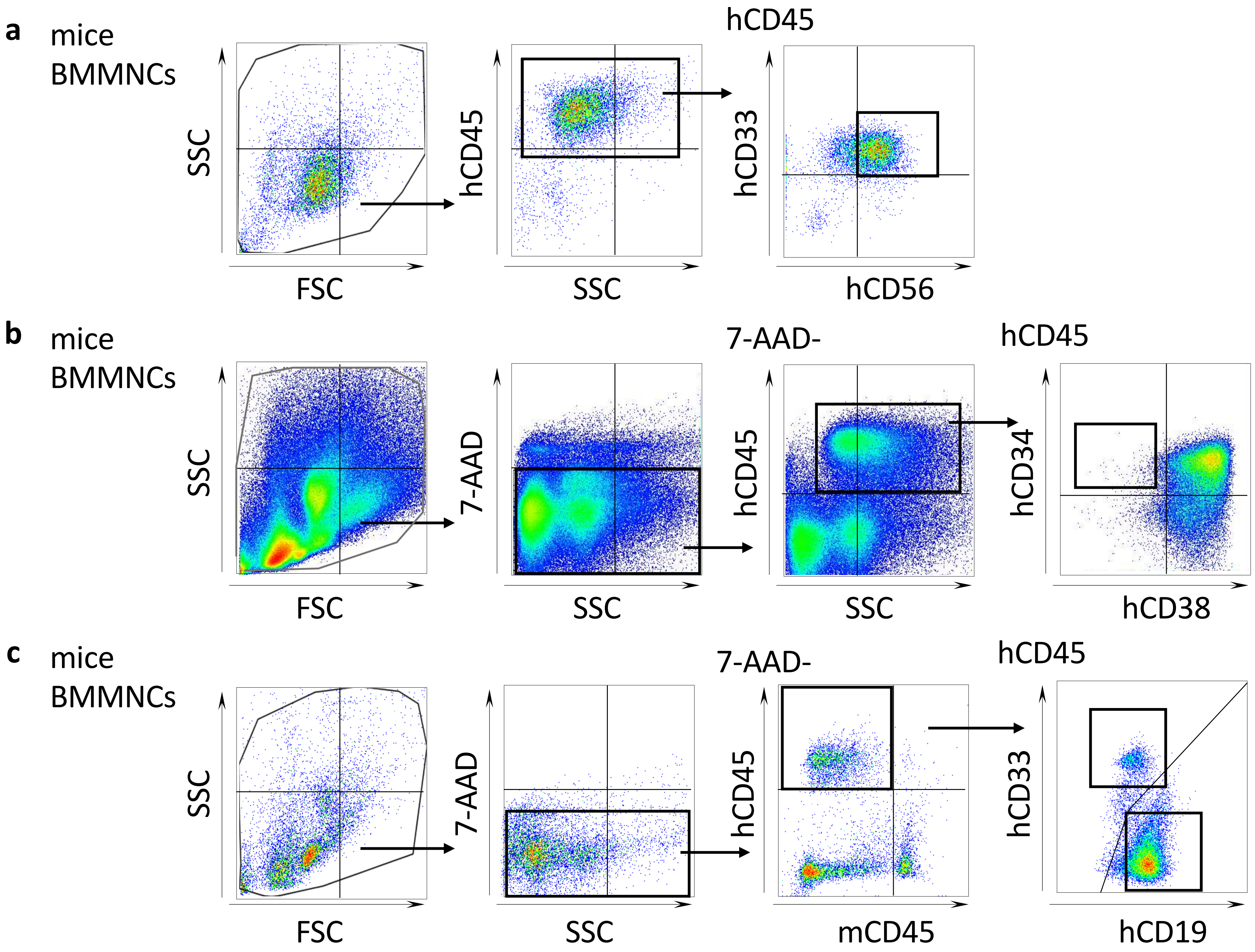
a
mice
BMMNCs
SSC
FSC
hCD45
SSC
hCD45
hCD33
hCD56
b
mice
BMMNCs
SSC
FSC
7-AAD
SSC
7-AAD-
hCD45
SSC
hCD45
hCD34
hCD38
c
mice
BMMNCs
SSC
FSC
7-AAD
SSC
7-AAD-
hCD45
mCD45
hCD45
hCD33
hCD19

Supplementary Figure 8

Gating strategies used for cell sorting and analysis with FACS

(a) Gating strategy with flowcytometry for MNCs from mice BM in Fig. 6b.

(b) Sorting and analysis with flowcytometry for MNCs from transplanted mice BM in supplementary Fig. 3.

(c) Gating strategy with flowcytometry for MNCs from mice BM in supplementary Fig. 7. For each color, cut offs for gating were based on comparing unstained, single stains, and fluorescence minus one strategy. Dead cells were detected by 7-AAD staining.

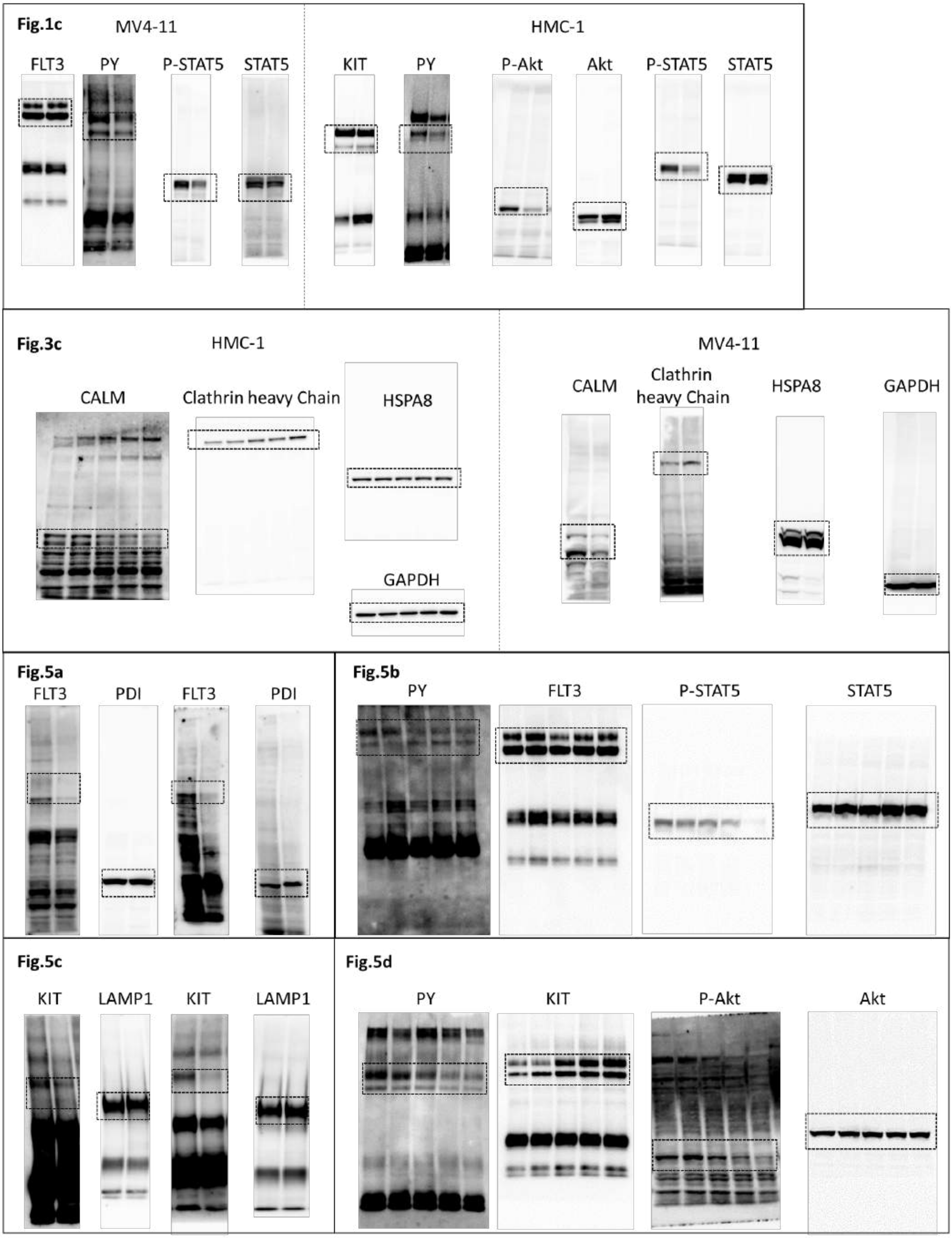
Fig.1c
MV4-11
HMC-1
FLT3
PY
P-STAT5
STAT5
KIT
PY
P-Akt
Akt
P-STAT5
STAT5
Fig.3c
HMC-1
MV4-11
CALM
Clathrin heavy Chain
HSPA8
GAPDH
CALM
Clathrin heavy Chain
HSPA8
GAPDH
Fig.5a
FLT3
PDI
FLT3
PDI
Fig.5b
PY
FLT3
P-STAT5
STAT5
Fig.5c
KIT
LAMP1
KIT
LAMP1
Fig.5d
PY
KIT
P-Akt
Akt

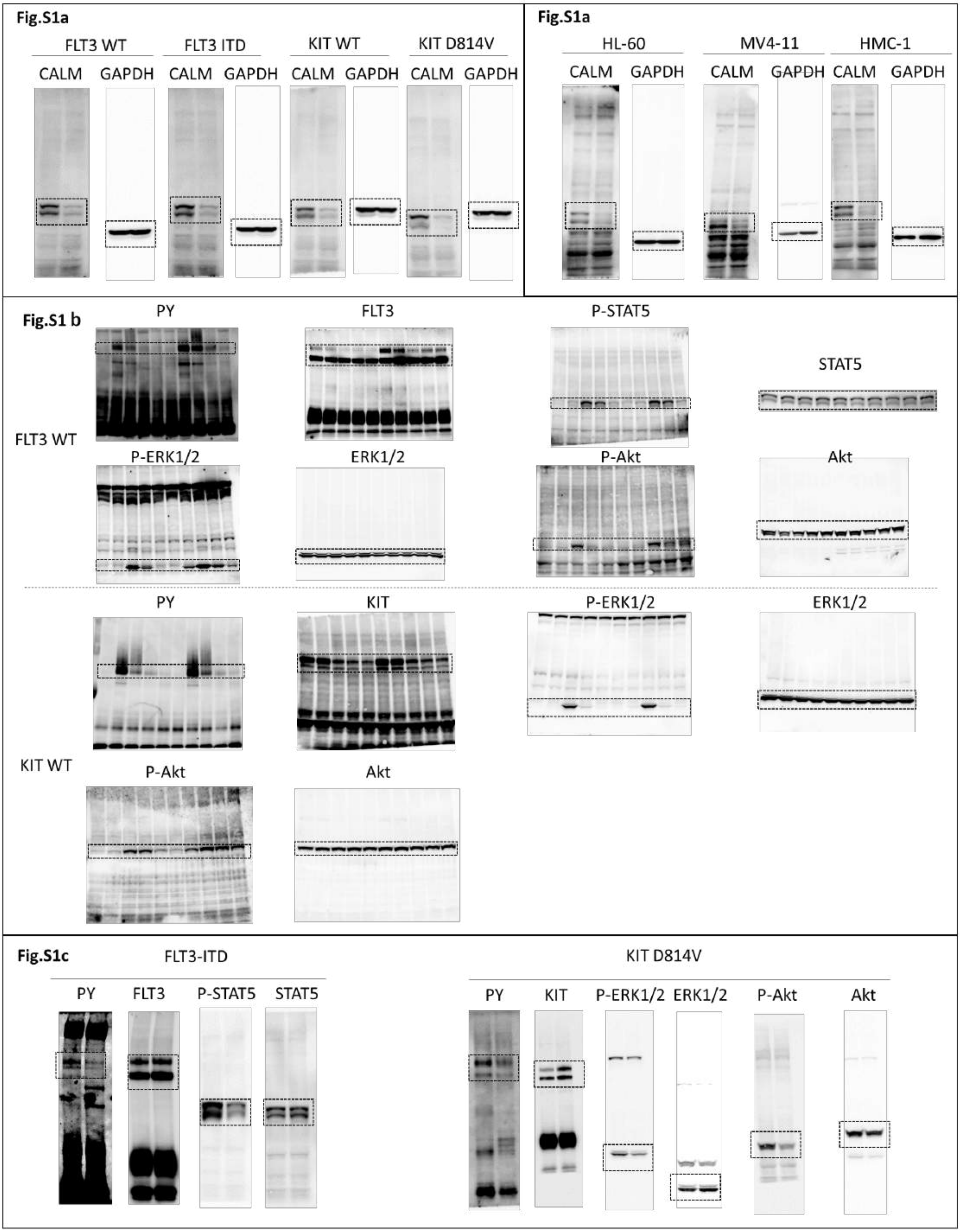
Fig.S1a
FLT3 WT
FLT3 ITD
KIT WT
KIT D814V
CALM
GAPDH
CALM
GAPDH
CALM
GAPDH
CALM
GAPDH
Fig.S1a
HL-60
MV4-11
HMC-1
CALM
GAPDH
CALM
GAPDH
CALM
GAPDH
Fig.S1 b
PY
FLT3
P-STAT5
STAT5
FLT3 WT
P-ERK1/2
ERK1/2
P-Akt
Akt
PY
KIT
P-ERK1/2
ERK1/2
KIT WT
P-Akt
Akt
Fig.S1c
FLT3-ITD
PY
FLT3
P-STAT5
STAT5
KIT D814V
PY
KIT
P-ERK1/2
ERK1/2
P-Akt
Akt

Supplementary Figure 9 and 9 contnd.

Uncropped versions of immunoblots analyses.

Supplementary Table 1

Clinical characteristics of patients.

Sample ID	Information at the time of sampling			
	Diagnosis	Cytogenetics	FLT3	c-KIT
1	M2	unknown	FLT3-ITD	wild type
2	M5b	normal karyotype	FLT3-ITD	wild type
3	M2	unknown	FLT3-ITD	wild type
4	M1	unknown	FLT3-ITD	wild type
5	M5b	del9(q),t(2;12)(p21;q13)	FLT3-ITD	wild type
6	M5b	normal karyotype	FLT3-ITD	wild type
7	M0	normal karyotype	FLT3-ITD	wild type
8	M2	normal karyotype	FLT3-ITD	wild type
10	M2	t(8;21)(q22;q22)	wild type	D816V
11	M2	t(8;21)(q22;q22)	wild type	wild type
12	M2	der(1;7)(q10;p10),der(18;21(q10;q10),del(20)(q11.2q13.3)	wild type	wild type
13	M2	normal karyotype	wild type	wild type
14	M2	t(8;21)(q22;q22)	wild type	wild type
15	M2	normal karyotype	wild type	wild type
16	M1	normal karyotype	wild type	wild type
17	M1	normal karyotype	wild type	wild type
18	M5b	t(6;11)(q27;q23)	wild type	wild type
19	M5b	t(11;19)(q23;p13.1)	wild type	wild type
20	M5b	normal karyotype	wild type	wild type

AML subtypes were determined according to the French-American-British classification.

Supplementary Table 2

List of antibodies used in this study.

	antibody	clone	company
Immunofluorescence analysis	biotinylated anti-CD117	104D2	Biolegend (San Diego, CA)
	biotinylated anti-CD135	BV10A4H2	
	anti-LAMP1	D2D11	Cell Signaling Technology (Danvers, MA)
	anti-GM130	D6B1	
	anti-PDI	C81H6	
	anti-EEA1	C45B10	
	anti-Rab11	D4F5	
	and anti-PDI AlexaFluor® 488-conjugated	C81H6	
	anti-CALM	A-2	Santa Cruz (Dallas, TX)
	AlexaFluor® 488-conjugated Goat anti-rabbit IgG		Thermo Fisher Scientific (Waltham, MA)
	AlexaFluor® 488-conjugated Goat anti-mouse IgG		
	AlexaFluor® 546-conjugated Goat anti-mouse IgG		
	AlexaFluor® 568 streptavidin conjugates		
	DAPI Fluoromount-G		Southern Biotech (Melbourne, Australia)
Immunohistochemistry analysis	Abs: anti-human CD56	1B6	Histofine, Nichirei Bio (Tokyo, Japan)
	anti-human LCA	2B11	
	anti-human CD34	NU-4A1	
	MAX-PO	MULTI	
Flow cytometry analysis	fluorescein isothiocyanate (FITC)-conjugated anti-human CD19	SJ25C1	BD Biosciences (Franklin Lakes, NJ)
	fluorescein isothiocyanate (FITC)-conjugated anti-human CD56	B159	
	phycoerythirin (PE)-conjugated anti-human CD33	WM53	
	phycoerythirin (PE)-conjugated anti-human CD38	HIT2	
	phycoerythirin (PE)-conjugated anti-mouse CD117	2B8	
	phycoerythirin (PE)-conjugated anti-mouse CD135	A2F10	Biolegend (San Diego, CA)
	PE-cyanin (cy)7-conjugated anti-human CD45	HI30	eBioscience (San Diego, CA)
	PE-cyanin (cy)7-conjugated anti-mouse CD45	30F11	
	allophycocyanin (APC)-conjugated anti-human CD34	8G12	BD Biosciences (Franklin Lakes, NJ)
Immunoblotting analysis	anti-CALM	A-2	Santa Cruz (Dallas, TX)
	anti-Actin	C-11	
	anti-phosphotyrosine	4G10	Cell Signaling Technology (Danvers, MA)
	anti-KIT	D13A2	
	anti-FLT3	8F2	
	anti-phosphorylated Akt (Ser473)	587F11	
	anti-Akt	C67E7	
	anti-phosphorylated p-44/42MAPK (T202/Y204)	20G11	
	anti-p44/42MAPK (ERK1/2)	137F5	
	anti-phosphorylated stat5	Y694	
	anti-stat5	3H7	
	anti-pan-Cadherin	#28E12	
	anti-HSP90	E289	
	anti-PDI	C81H6	
	anti-LAMP1	D2D11	
	anti-HSPA8	D12F2	
	anti-Clathrin Heavy Chain	D3C6	
