# Supplementary material for: Chlorpromazine eliminates acute myeloid leukemia cells by perturbing subcellular localization of FLT3-ITD and KIT-D816V
Source: Nat Commun. 2020 Aug 18;11:4147. doi: 10.1038/s41467-020-17666-8 (PMC7434901; doi:10.1038/s41467-020-17666-8)
Supplement: Supplementary file 3 — Reporting summary [file 41467_2020_17666_MOESM3_ESM.pdf]

natureresearch

Corresponding author(s): Hirokazu Tanaka, M.D., Ph.D.

Last updated by author(s): Jun 26, 2020

Reporting Summary

Nature Research wishes to improve the reproducibility of the work that we publish. This form provides structure for consistency and transparency in reporting. For further information on Nature Research policies, see Authors & Referees and the Editorial Policy Checklist .

Statistics

For all statistical analyses, confirm that the following items are present in the figure legend, table legend, main text, or Methods section.

n/a	Confirmed	
☐	☒	The exact sample size (*n*) for each experimental group/condition, given as a discrete number and unit of measurement
☐	☒	A statement on whether measurements were taken from distinct samples or whether the same sample was measured repeatedly
☐	☒	The statistical test(s) used AND whether they are one- or two-sided<br>*Only common tests should be described solely by name; describe more complex techniques in the Methods section.*
☒	☐	A description of all covariates tested
☒	☐	A description of any assumptions or corrections, such as tests of normality and adjustment for multiple comparisons
☐	☒	A full description of the statistical parameters including central tendency (e.g. means) or other basic estimates (e.g. regression coefficient) AND variation (e.g. standard deviation) or associated estimates of uncertainty (e.g. confidence intervals)
☐	☒	For null hypothesis testing, the test statistic (e.g. *F*, *t*, *r*) with confidence intervals, effect sizes, degrees of freedom and *P* value noted<br>*Give P values as exact values whenever suitable.*
☒	☐	For Bayesian analysis, information on the choice of priors and Markov chain Monte Carlo settings
☒	☐	For hierarchical and complex designs, identification of the appropriate level for tests and full reporting of outcomes
☒	☐	Estimates of effect sizes (e.g. Cohen's *d*, Pearson's *r*), indicating how they were calculated

Our web collection on statistics for biologists contains articles on many of the points above.

Software and code

Policy information about availability of computer code

Data collection	All software used for data collection is commercially available and stated in the Methods section.<br><br>Viable cell number was evaluated by Envision plate reader (Wallac, 1420 ARVO MX-2, Turku, Finland).<br>Densitometric analysis of western blot imaging was performed by Image Quant TL (GE Healthcare) (Version 8.1).<br>Flow cytometry was performed by FACS Canti-II and FACS Aria-II (BD Biosciences, Franklin Lakes, NJ).<br>Immunofluorescence analysis was performed by BZ-X710 All-in-One fluorescence microscope (Keyence Corp, Osaka, Japan).<br>PCR analysis was performed using GeneAmp PCR system 9700 (Thermo Fisher Scientific).<br>Sequence analysis was performed using genetic analyzer 3100 (Thermo Fisher Scientific).<br>Immunohistochemical images were taken using a clinical microscope instrument (Eclipse-Ci, Nikon, Tokyo, Japan) equipped with a digital camera (DS-Ril, Nikon).
Data analysis	All other software used for data analysis is detailed in the Methods section.<br><br>The flow cytometry data were analyzed by Flow Jo software (Tree Star, USA) (Version 9.7.7).<br>The co-localizations were measured via Manders' Colocalization Coefficients using ImageJ software (NIH, Bethesda, MD)(Version 1.51).<br>Statistical analysis was performed using the Graph Pad prism 8 software package (San Diego, CA, USA)(Version 8).

For manuscripts utilizing custom algorithms or software that are central to the research but not yet described in published literature, software must be made available to editors/reviewers. We strongly encourage code deposition in a community repository (e.g. GitHub). See the Nature Research guidelines for submitting code & software for further information.

Data

Policy information about availability of data

All manuscripts must include a data availability statement. This statement should provide the following information, where applicable:

- Accession codes, unique identifiers, or web links for publicly available datasets
- A list of figures that have associated raw data
- A description of any restrictions on data availability

The source data underlying Figs 1a-c, 2a-e, 3 a-e, 4 c, f, 5 a-d, 6 c and Supplementary Figs are provided as a Source Data file. The data that support the findings of this study are available from the corresponding author upon reasonable request.

Field-specific reporting

Please select the one below that is the best fit for your research. If you are not sure, read the appropriate sections before making your selection.

☒ Life sciences ☐ Behavioural & social sciences ☐ Ecological, evolutionary & environmental sciences

For a reference copy of the document with all sections, see nature.com/documents/nr-reporting-summary-flat.pdf

Life sciences study design

All studies must disclose on these points even when the disclosure is negative.

Sample size	Sample size was decided by previously published results and our experience with the techniques in this research. All key experiments were repeated independently using different cell lines and clones or different techniques. From our statistic results, we were confident that the sample size we used was sufficient to support our results. For human samples, sample size was determined by the availability of samples. The exact numbers of animal and human samples are indicated in each figure.
Data exclusions	No data were excluded from the analysis.
Replication	All the experimental findings were reliably reproduced.The exact numbers are clearly indicated in the figure legends. All attempts at replication were successful.
Randomization	The animals were randomly assigned to each experimental/vehicle group determined by the availability of samples (3-6 mice/group). All samples or mice were included in our analysis.
Blinding	Investigators were not blinded in this study because no clinical relevant experiments were performed. However, there is no bias for all the data collected in this study.

Reporting for specific materials, systems and methods

We require information from authors about some types of materials, experimental systems and methods used in many studies. Here, indicate whether each material, system or method listed is relevant to your study. If you are not sure if a list item applies to your research, read the appropriate section before selecting a response.

Materials & experimental systems

n/a	Involved in the study
☐	☒ Antibodies
☐	☒ Eukaryotic cell lines
☒	☐ Palaeontology
☐	☒ Animals and other organisms
☐	☒ Human research participants
☒	☐ Clinical data

Methods

n/a	Involved in the study
☒	☐ ChIP-seq
☐	☒ Flow cytometry
☒	☐ MRI-based neuroimaging

Antibodies

Antibodies used

	the supplier name	catalog number	dilution
Immunofluorescence analysis:√ √ √ √ √ √ √ √			
biotinylated anti-CD117 (104D2)	Biolegend	313208	*100
biotinylated anti-CD135 (BV10A4H2)	Biolegend	313312	*100
anti-LAMP1 (D2D11)	CST√ √ √	9091√ √	*100
anti-GM130 (D6B1)	CST√ √	12480√ √	*3200
anti-PDI (C81H6)	CST√ √	3501√ √	*50

anti-EEA1 (C45B10)	CST	3288	*100
anti-Rab11 (D4F5)	CST	5589	*50
anti-CALM (A-2)	Santa Cruz	sc-6433	*50
AlexaFluor® 488-conjugated Goat anti-rabbit IgG	Thermo Fisher	A11034	*400
AlexaFluor® 488-conjugated Goat anti-mouse IgG	Thermo Fisher	A11001	*400
AlexaFluor® 546-conjugated Goat anti-mouse IgG	Thermo Fisher	A11030	*100
AlexaFluor® 568 streptavidin conjugates	Thermo Fisher	S11226	*100
DAPI Fluoromount-G	Southern Biotech	0100-20	ready-to-use
Immunohistochemistry analysis:			
anti-human CD56 (1B6)	NICHIREI	413331	ready-to-use
anti-human LCA (2B11)	NICHIREI	422071	ready-to-use
anti-human CD34 (NU-4A1)	NICHIREI	413111	ready-to-use
MAX-PO MULTI	NICHIREI	424152	ready-to-use
Flow cytometry analysis:			
FITC-conjugated anti-human CD19 (SJ25C1)	BD	340409	*40
FITC-conjugated anti-human CD56 (B159)	BD	340410	*40
PE-conjugated anti-human CD33 (WM53)	BD	347787	*40
PE-conjugated anti-human CD38 (HB7)	BD	347687	*40
PE -conjugated anti-mouse CD117 (2B8)	BD	553355	*40
PE -conjugated anti-mouse CD135 (A2F10)	Biolegend	135306	*40
PE-cy7-conjugated anti-human CD45 (HI30)	e-Bioscience	25-0459-41	*50
PE-cy7-conjugated anti-mouse CD45 (30F11)	Biolegend	103113	*50
APC-conjugated anti-human CD34 (8G12)	BD	340441	*50
Immunoblotting analysis:			
anti-CALM (A-2)	Santa Cruz	sc-6433	*50
anti-Actin (C-11)	Santa Cruz	sc-1615	*1000
anti-phosphotyrosine (4G10)	MILLIPORE	05-1050	*1000
anti-KIT (D13A2)	CST	3074	*1000
anti-FLT3 (8F2)	CST	3462	*500
anti-phosphorylated Akt (S473)(193H12)	CST	4058	*1000
anti-Akt (C67E7)	CST	4691	*1000
anti-phosphorylated p-44/42MAPK (T202/Y204) (20G11)	CST	4376	*1000
anti-p44/42MAPK (ERK1/2) (137F5)	CST	4695	*1000
anti-phosphorylated stat5 (Y694)	CST	9359	*1000
anti-stat5 (3H7)	CST	9358	*1000
anti-pan-Cadherin (28E12)	CST	4073	*1000
anti-HSP90 (E289)	CST	4875	*1000
anti-PDI (C81H6)	CST	3501	*1000
anti-LAMP1 (D2D11)	CST	9091S	*1000
anti-HSPA8 (D12F2)	CST	8444S	*1000
anti-Clathrin Heavy Chain (D3C6)	CST	4796S	*1000

Validation

All the antibodies used in the flow cytometry experiments, immunofluorescence analyses, and immunoblot and immunoprecipitation assay were from commercial vendors and they were validated for specificity to original targets by the manufacturers. Antibody validation by the manufacturer is available at each manufacturer's website by searching under the provided antibody part numbers.

Eukaryotic cell lines

Policy information about cell lines

Cell line source(s)

BALL-1, HL-60, TALL-1, KG-1, THP-1, Kasumi, and K562 were purchased from Japanese Collection of Research Bioresources Cell Bank (National Institutes of Biomedical Innovation, Health and Nutrition, Japan). HMC-1, MV4-11, Ba/F3 and 293T cells were purchased from American Tissue Culture Collection (Manassas, VA).

Authentication

The cell lines were not authenticated.

Mycoplasma contamination

The cell lines were not tested for mycoplasma contamination.

Commonly misidentified lines
(See ICLAC register)

None of the used cell lines are listed in the database of commonly misidentified cell lines.

Animals and other organisms

Policy information about studies involving animals; ARRIVE guidelines recommended for reporting animal research

Laboratory animals	Six to eight-week old female BALB/C mice and nude mice (BALB/cAJcl-nu/nu mice) purchased from CLEA Japan, Inc., Tokyo, Japan were used for transplantation assay of Ba/F3 FLT3 ITD cells, and Ba/F3 KIT D814V cells, respectively. For xenotransplantation of primary AML cells, 6-8-week old female NOD/Scid/IL2Rgamma-KO (NOG) mice were ordered from Central Institute for Experimental Animals (Kawasaki, Kanagawa, Japan). All mice were bred and maintained under specific-pathogen-free conditions at the animal facilities of Kindai University, and animals are kept in groups of 5-6 in individually ventilated caging system and provided continuously with sterile water and chow pellets. The intra-cage temperature was 23±1°C and the relative humidity 50 ±10%. A 12:12 Dark-Light cycle was operating in the room.
Wild animals	No wild animals used in this study.
Field-collected samples	The study did not involve field-collected samples.
Ethics oversight	The study is compliant with all relevant ethical regulations for animal experiments. All animal experiments were conducted after getting the approval from the committee of animal experiments in Kindai university (Approval ID 06-13).

Note that full information on the approval of the study protocol must also be provided in the manuscript.

Human research participants

Policy information about studies involving human research participants

Population characteristics	Detailed information of human material used in this study are provided in Suppl. Table 1.<br>Samples were flash frozen and stored at -80°C. BMMNCs containing >70% AML cells were isolated from patients with the indicated status of FLT3 and KIT genes.
Recruitment	Patients were not actively recruited. De novo AML patient residual samples with or without FLT3 ITD or KIT mutations were obtained after written informed consent following institutional guidelines of Kindai University Faculty of Medicine (Authorization Number:24-017, -018) per Declaration of Helsinki principles.
Ethics oversight	All experiments were conducted after getting the approval from Ethics Committee of Kindai University Faculty of Medicine (Approval ID 29-072).

Note that full information on the approval of the study protocol must also be provided in the manuscript.

Flow Cytometry

Plots

Confirm that:

- [x] The axis labels state the marker and fluorochrome used (e.g. CD4-FITC).
- [x] The axis scales are clearly visible. Include numbers along axes only for bottom left plot of group (a 'group' is an analysis of identical markers).
- [x] All plots are contour plots with outliers or pseudocolor plots.
- [x] A numerical value for number of cells or percentage (with statistics) is provided.

Methodology

Sample preparation	For flow cytometry analysis from cell populations in bone marrow, femur and tibia were isolated and sterilised with 70% ethanol. The bones were cut open and were flushed with cold 1X PBS using a syringe. The cells were filtered through a 40um cell strainer and washed with cold 1X PBS.<br>Before labelling the cells with antibodies for flow cytometry, the erythrocytes were eliminated from the samples by incubating with 3-5 ml erythrocyte lysis buffer. Then cells were treated with blocking IgG (BD Fc Block Reagent) for 10 min at RT.<br>The reaction was stopped by adding 1X PBS and the cells were washed by centrifugation at 400xg for 10 min at 4°C.<br>For flow cytometry, 1 x 10e5 - 1 x 10e6 cells were suspended in 100ul of cold 1X PBS in a FACS tube.
Instrument	BD FACS Canto II was used for analysis and BD FACS Aria II sorter was used for sorting.
Software	The flow cytometry data was collected by BD FACS DIVA software. All flow cytometry data were analyzed by Flow Jo software version 7.6.5 and version 10.2 (Tree Star, USA)
Cell population abundance	The relevant cell populations are sorted using BD FACS ARIA II. The cell purity was analyzed by flow cytometry by comparing the cell populations before and after sorting. All the post-sort fractions were at least 90% pure.

Gating strategy

For each color, cut offs for gating were based on comparing unstained, single stains, and fluorescence minus one strategies.
Dead cells were detected by 7-AAD staining.
The strategy is depicted in Suppl. Fig. 8 and has been applied for all FACS analyses.

☒ Tick this box to confirm that a figure exemplifying the gating strategy is provided in the Supplementary Information.
